# Supplementary material for: Caffeine Intake Alters Recovery Sleep after Sleep Deprivation
Source: Nutrients. 2024 Oct 11;16(20):3442. doi: 10.3390/nu16203442 (PMC11510014; doi:10.3390/nu16203442)
Supplement: Supplementary file 1 [file nutrients-16-03442-s001.zip › nutrients-3249092-supplementary.pdf]

# Caffeine intake alters recovery sleep after sleep deprivation

## *Supplementary data*

***Table S1. Mixed linear model results for sleep parameters***

| | Effects | F | Degrees of freedom (Df)) | | P |
|---|---|---|---|---|---|
| **TST** | Treatment (TTT) | 6.31 | 1 | 70.5 | **0.01** |
| | Night | 197.64 | 1 | 35.9 | **0.001** |
| | Caff. habitual consumption | 3.94 | 1 | 65.3 | 0.08 |
| | TTT x Night | 3.65 | 1 | 70.0 | **0.04** |
| | TTT x Caff. habitual consumption | 0.64 | 1 | 102.2 | 0.42 |
| | Night x Caff. habitual consumption | 3.94 | 1 | 95.4 | **0.04** |
| | TTT x Night x Caff. habitual consumption | 0.750 | 1 | 101.9 | 0.39 |
| **N1** | Treatment (TTT) | 1.89 | 1 | 93.9 | 0.17 |
| | Night | 9.68 | 1 | 93.5 | **0.002** |
| | Caff. habitual consumption | 2.50 | 1 | 118.4 | 0.11 |
| | TTT x Night | 0.07 | 1 | 93.9 | 0.78 |
| | TTT x Caff. habitual consumption | 0.11 | 1 | 119.6 | 0.73 |
| | Night x Caff. Habitual consumption | 0.27 | 1 | 114.9 | 0.60 |
| | TTT x Night x Caff. Habitual Consumption | 0.51 | 1 | 118.2 | 0.47 |
| **N2** | Treatment (TTT) | 0.08 | 1 | 97.9 | 0.77 |
| | Night | 2.17 | 1 | 97.6 | 0.14 |
| | Caff. habitual consumption | 0.01 | 1 | 131.7 | 0.75 |
| | TTT x Night | 0.27 | 1 | 97.9 | 0.60 |
| | TTT x Caff. habitual Consumption | 1.17 | 1 | 110.4 | 0.28 |
| | Night x Caff. habitual consumption | 0.001 | 1 | 110.1 | 0.94 |
| | TTT x Night x Caff. habitual consumption | 0.31 | 1 | 109.6 | 0.57 |
| **N3** | Treatment (TTT) | 0.98 | 1 | 128.71 | 0.32 |
| | **Night** | **30.28** | **1** | **100.52** | **0.001** |
| | Caff. habitual consumption | 2.16 | 1 | 100.49 | 0.14 |
| | **TTT x Night** | **4.22** | **1** | **100.47** | **0.04** |
| | TTT x Caff. habitual cConsumption | 2.64 | 1 | 105.18 | 0.10 |
| | Night x Caff. habitual cConsumption | 0.03 | 1 | 105.26 | 0.85 |
| | TTT x Night x Caff. habitual consumption | 0.11 | 1 | 104.56 | 0.74 |
| **REM** | Treatment (TTT) | 1.01 | 1 | 132.00 | 0.316 |
| | Night | **13.27** | **1** | **104.06** | **0.001** |
| | Caff. habitual consumption | 1.12 | 1 | 104.62 | 0.292 |
| | TTT x Night | 0.06 | 1 | 104.60 | 0.804 |
| | TTT x Caff. habitual consumption | 5.10 | 1 | 109.85 | 0.026 |
| | Night x Caff. habitual consumption | 1.77 | 1 | 111.08 | 0.186 |
| | TTT x Night x Caff. habitual consumption | 0.04 | 1 | 110.17 | 0.846 |

***Table S2. Mixed linear model results for sleep parameters, in % TST***

| | **Effects** | **F** | **Degrees of freedom (Df))** | | **P** |
|---|---|---|---|---|---|
| **N1 (%TST)** | Treatment (TTT) | 1.76 | 1 | 93.91 | 0.18 |
| | **Night** | **11.59** | **1** | **93.51** | **0.001** |
| | Caff. habitual consumption | 2.75 | 1 | 118.41 | 0.10 |
| | TTT x Night | 0.14 | 1 | 93.92 | 0.71 |
| | TTT x Caff. habitual consumption | 0.07 | 1 | 119.61 | 0.79 |
| | Night x Caff. habitual consumption | 0.56 | 1 | 114.92 | 0.45 |
| | TTT x Night x Caff. habitual consumption | 0.49 | 1 | 118.21 | 0.48 |
| **N2 (%TST)** | Treatment (TTT) | 0.00 | 1 | 97.93 | 0.98 |
| | **Night** | **9.16** | **1** | **97.62** | **0.003** |
| | Caff. habitual consumption | 0.09 | 1 | 131.74 | 0.76 |
| | TTT x Night | 0.75 | 1 | 97.91 | 0.39 |
| | TTT x Caff. habitual consumption | 1.17 | 1 | 110.43 | 0.28 |
| | Night x Caff. habitual consumption | 0.29 | 1 | 110.1 | 0.59 |
| | TTT x Night x Caff. habitual consumption | 0.47 | 1 | 109.61 | 0.49 |
| **N3 (%TST)** | Treatment (TTT) | 3.60 | 1 | 97.92 | 0.06 |
| | **Night** | **46.36** | **1** | **97.63** | **0.001** |
| | Caff. habitual consumption | 0.90 | 1 | 131.74 | 0.34 |
| | **TTT x Night** | **5.02** | **1** | **97.91** | **0.02** |
| | TTT x Caff. habitual consumption | 0.02 | 1 | 110.42 | 0.89 |
| | Night x Caff. habitual consumption | 3.53 | 1 | 110.13 | 0.06 |
| | TTT x Night x Caff. habitual consumption | 0.24 | 1 | 109.61 | 0.62 |
| **REM (%TST)** | Treatment (TTT) | 0.25 | 1 | 97.94 | 0.62 |
| | Night | 0.06 | 1 | 97.68 | 0.80 |
| | Caff. habitual consumption | 0.61 | 1 | 131.78 | 0.43 |
| | TTT x Night | 0.19 | 1 | 97.92 | 0.66 |
| | TTT x Caff. habitual consumption | 2.32 | 1 | 110.49 | 0.13 |
| | **Night x Caff. habitual consumption** | **4.71** | **1** | **110.11** | **0.03** |
| | TTT x Night x Caff. habitual consumption | 0.02 | 1 | 109.62 | 0.88 |

*Caff. is caffeine*

***Table S3. Mixed linear model results for sleep latencies***

| | Effects | F | Degrees of freedom (Df)) | | P |
|---|---|---|---|---|---|
| **Sleep Onset Latency (SOL)** | Treatment (TTT) | 0.82 | 1 | 103.78 | 0.36 |
| | **Night** | **11.83** | **1** | **103.78** | **0.001** |
| | Caff. habitual consumption | 0.01 | 1 | 109.94 | 0.94 |
| | TTT x Night | 1.35 | 1 | 103.78 | 0.24 |
| | TTT x Caff. habitual consumption | 3.61 | 1 | 125.10 | 0.06 |
| | Night x Caff. habitual consumption | 3.01 | 1 | 125.33 | 0.09 |
| | TTT x Night x Caff. habitual consumption | 1.02 | 1 | 125.10 | 0.32 |
| **N2 Latency** | Treatment (TTT) | 0.68 | **1** | 103.78 | 0.41 |
| | Night | 2.93 | **1** | 103.79 | 0.09 |
| | Caff. habitual consumption | 0.00 | **1** | 109.94 | 0.98 |
| | TTT x Night | 0.02 | **1** | 103.78 | 0.90 |
| | TTT x Caff. habitual consumption | 1.34 | **1** | 125.10 | 0.24 |
| | Night x Caff. habitual consumption | 1.26 | **1** | 125.33 | 0.26 |
| | TTT x Night x Caff. habitual consumption | 0.23 | **1** | 125.10 | 0.63 |
| **N3 Latency** | Treatment (TTT) | 1.94 | 1 | 103.78 | 0.16 |
| | **Night** | **57.96** | **1** | 103.79 | **0.001** |
| | Caff. habitual consumption | 0.22 | 1 | 109.94 | 0.64 |
| | TTT x Night | 0.86 | 1 | 103.78 | 0.51 |
| | TTT x Caff. habitual consumption | 3.18 | 1 | 125.10 | 0.07 |
| | Night x Caff. habitual consumption | 1.47 | 1 | 125.33 | 0.18 |
| | TTT x Night x Caff. habitual consumption | 0.03 | 1 | 125.10 | 0.85 |
| **REM latency** | Treatment (TTT) | 0.57 | 1 | 103.78 | 0.45 |
| | **Night** | **5.91** | **1** | 103.79 | **0.02** |
| | Caff. habitual consumption | 0.20 | 1 | 109.94 | 0.65 |
| | TTT x Night | 0.07 | 1 | 103.78 | 0.76 |
| | TTT x Caff. habitual consumption | 0.24 | 1 | 125.10 | 0.62 |
| | Night x Caff. habitual consumption | 3.46 | 1 | 125.33 | 0.06 |
| | TTT x Night x Caff. habitual consumption | 0.40 | 1 | 125.10 | 0.52 |

*Caff. is caffeine*

***Table S4. Mixed linear model results for sleep quality***

| | Effects | F | Degrees of freedom (Df)) | | P |
|---|---|---|---|---|---|
| **WASO (min)** | Treatment (TTT) | 0.67 | 1 | 78.2 | 0.41 |
| | Night | 0.16 | 1 | 100.7 | 0.68 |
| | Caff. habitual consumption | 2.98 | 1 | 92.5 | 0.08 |
| | TTT x Night | 0.27 | 1 | 104.9 | 0.60 |
| | TTT x Caff. habitual consumption | 1.79 | 1 | 104.9 | 0.18 |
| | Night x Caff. habitual consumption | 2.32 | 1 | 100.0 | 0.13 |
| | TTT x Night x Caff. habitual consumption | 1.16 | 1 | 109.6 | 0.28 |
| **WASO (n)** | Treatment (TTT) | 0.57 | 1 | 78.2 | 0.40 |
| | Night | 3.16 | 1 | 100.7 | 0.18 |
| | Caff. habitual consumption | 3.98 | 1 | 92.5 | 0.07 |
| | **TTT x Night** | **5.31** | **1** | **104.9** | **0.04** |
| | **TTT x Caff. habitual consumption** | **5.79** | **1** | **104.9** | **0.04** |
| | Night x Caff. habitual consumption | 2.22 | 1 | 100.0 | 0.11 |
| | TTT x Night x Caff. habitual consumption | 3.26 | 1 | 109.6 | 0.12 |
| **SEI (%)** | Treatment (TTT) | 0.14 | 1 | 103.66 | 0.71 |
| | **Night** | **4.60** | **1** | **103.66** | **0.03** |
| | Caff. habitual consumption | 0.33 | 1 | 124.05 | 0.56 |
| | TTT x Night | 0.02 | 1 | 103.66 | 0.88 |
| | TTT x Caff. habitual consumption | 3.03 | 1 | 121.00 | 0.08 |
| | Night x Caff. habitual consumption | 1.04 | 1 | 122.02 | 0.30 |
| | TTT x Night x Caff. habitual consumption | 0.27 | 1 | 121.00 | 0.60 |

*Caff. is caffeine; SEI is sleep efficiency index*
